# Low Anterior Resection Syndrome: Current Understanding and Management

**DOI:** 10.7759/cureus.83942

**Published:** 2025-05-12

**Authors:** Supratim Bhattacharyya, Antarip Bhattacharya, Prosenjit Das, Amit Choraria

**Affiliations:** 1 Surgical Oncology, Apollo Hospitals, Kolkata, IND; 2 General Surgery, Newham University Hospital, Barts Health NHS Trust, London, GBR

**Keywords:** bowel dysfunction, colorectal surgery, fecal incontinence, lars, low anterior resection syndrome, pelvic floor rehabilitation, rectal cancer, sacral nerve stimulation, sphincter-preserving surgery, total mesorectal excision

## Abstract

Low anterior resection syndrome (LARS) is a common and often debilitating complication following sphincter-preserving surgery for rectal cancer. With evolving techniques in surgical oncology, sphincter preservation has become more prevalent; however, this has led to an increased incidence of postoperative bowel dysfunction. This narrative review explores the current understanding of LARS, including its definition, pathophysiology, clinical evaluation, treatment options, and preventive strategies. Emphasis is placed on both conservative and surgical management approaches, as well as the importance of patient education and multidisciplinary care in improving quality of life.

## Introduction and background

Colorectal cancer is the third most common cancer worldwide, with rectal cancer comprising nearly one-third of all colorectal malignancies [1]. Advances in multimodal treatment, including neoadjuvant chemoradiation, refined surgical techniques such as total mesorectal excision (TME), and enhanced recovery protocols have significantly improved local control and overall survival [2,3]. These innovations have also facilitated sphincter-preserving surgeries, such as low anterior resection (LAR) and intersphincteric resection (ISR), which reduce the need for permanent stomas and improve cosmetic and psychological outcomes.

However, these benefits come with a caveat: up to 80-90% of patients undergoing these procedures report varying degrees of postoperative bowel dysfunction, collectively termed "low anterior resection syndrome" (LARS) [4]. The syndrome affects physical comfort, emotional well-being, and social functioning, often leading to social withdrawal and reduced quality of life. As LARS becomes an increasingly recognised clinical entity, a deeper understanding of its aetiology, risk factors, assessment, and management becomes imperative.

## Review

Definition and classification of LARS

LARS is defined as disordered bowel function that occurs after rectal resection, resulting in a significant detriment to quality of life [5]. The syndrome encompasses a spectrum of symptoms, including faecal and gas incontinence, urgency, frequency, clustering of stools (repeated bowel movements over a short period), and a sensation of incomplete evacuation. Conversely, some patients may experience obstructive symptoms, such as constipation and difficulty in evacuation.

These symptoms may present in isolation or in combination. The condition is typically classified as short-term (resolving within 6-12 months post-surgery) or long-term (persisting beyond 12 months) [6]. The LARS score, a validated patient-reported questionnaire, is the most commonly used tool to quantify symptom severity and stratify patients into no LARS, minor LARS, or major LARS categories.

Differential diagnosis

Though LARS is a postoperative diagnosis, it can present with symptoms similar to other bowel conditions. Thus, when evaluating patients with symptoms suggestive of LARS, it is essential to consider other potential causes of bowel dysfunction. Conditions such as irritable bowel syndrome (IBS), chronic radiation proctitis, inflammatory bowel disease (IBD), small intestinal bacterial overgrowth (SIBO), and bile acid malabsorption can present with similar symptoms, including diarrhoea, urgency, and incontinence. Structural complications, such as anastomotic strictures and pelvic abscesses, should also be excluded through appropriate imaging and endoscopic evaluation. Thorough clinical assessment, including history, physical examination, and selective use of diagnostic tests such as colonoscopy, anorectal manometry, and hydrogen breath tests, is crucial to differentiate LARS from these conditions and avoid misdiagnosis.

Surgical anatomy and physiology of the anal sphincter complex

Understanding the normal anatomy and physiology of the anal sphincter complex is fundamental to comprehending the pathogenesis of LARS. The internal anal sphincter (IAS), a continuation of the circular smooth muscle of the rectum, contributes 55-75% of resting anal pressure [7,8]. This muscle is surrounded by longitudinal muscle fibres that extend into the pelvic floor and play a supportive role in defecation [9,10].

The external anal sphincter (EAS) consists of striated muscle fibres and is under voluntary control, contributing to the dynamic maintenance of continence. It encircles the IAS and is closely associated with the levator ani muscle, particularly the puborectalis, which forms a sling around the anorectal junction. This anatomical arrangement ensures that the anorectal angle is maintained, contributing significantly to continence.

Between the IAS and EAS lies the intersphincteric space, an important anatomical plane exploited during intersphincteric resection (ISR) in ultra-low rectal tumours [11,12]. The surgical approach through this space allows for tumour resection while preserving the EAS, provided the tumour has not invaded this plane.

Physiologically, defaecation begins when rectal distension triggers the rectoanal inhibitory reflex (RAIR), characterised by transient relaxation of the IAS, allowing discrimination between flatus and faeces [13,14]. If defecation is socially inappropriate, voluntary contraction of the EAS and the puborectalis muscle allows the stool to be retained. Disruption of this finely tuned mechanism through surgical trauma or nerve injury is a key contributor to the symptoms seen in LARS.

Pathophysiology of LARS

The pathogenesis of LARS is multifactorial. One of the key mechanisms is the loss of rectal reservoir function following resection. This significantly diminishes the capacity of the rectum to accommodate stool, thereby contributing to urgency and increased stool frequency. The neorectum, fashioned from the remaining colon, often lacks the compliance and sensory function of the native rectum, further impairing its function.

A primary contributor is nerve injury sustained during pelvic dissection, particularly in areas adjacent to the posterolateral aspect of the prostate, where autonomic nerves critical for anorectal function are located [15,16]. In addition, direct trauma or inadvertent stretching of the IAS during surgery can impair its function, contributing to incontinence and urgency [17]. Another significant factor is the reduction in neorectal compliance, often due to the creation of shorter rectal segments post-anastomosis, which compromises the rectum’s reservoir capacity [18,19]. The loss of the rectoanal inhibitory reflex (RAIR), a vital component of continence that allows for rectal content sampling, further disrupts anorectal coordination [20]. Moreover, a decrease in neorectal sensitivity can impair the patient’s ability to discern stool from gas, resulting in unpredictable bowel movements and soiling [21]. Lastly, hyperactive motility within the neorectum can lead to increased frequency and urgency of defecation [22,23]. These factors frequently coexist in varying combinations, resulting in a heterogeneous and patient-specific symptom profile that complicates both diagnosis and management.

Clinical evaluation: the LARS score

The LARS score is a validated and widely used clinical tool developed to objectively assess the severity of LARS and its impact on a patient’s quality of life. It is based on a brief questionnaire consisting of five key symptoms commonly experienced by patients following low anterior resection, as summarised in Table 1. These are incontinence for flatus, incontinence for liquid stools, frequency of bowel movements, clustering of bowel movements (i.e., repeated evacuations over a short time span), and urgency. Each of these items is assigned a weighted score, with the total ranging from 0 to 42.

**Table 1:** The LARS Score Questionnaire and Classification Score Interpretation: 0–20: No LARS, 21–29: Minor LARS, 30–42: Major LARS

Symptom	Response Option	Score
Incontinence for flatus	Never	0
	<1/month	4
	≥1/month	7
	Weekly	11
	Daily	13
Incontinence for liquid stool	Never	0
	<1/month	3
	≥1/month	5
	Weekly	7
	Daily	9
Frequency of bowel movements per day	≤3	0
	4–7	4
	≥8	7
Clustering (≥2 bowel movements per hour)	No	0
	Yes	11
Urgency	No	0
	Yes	16

This simple yet effective scoring system has demonstrated high sensitivity and specificity for identifying patients who experience significant postoperative bowel dysfunction. The LARS score is not only a clinical diagnostic tool, but it also aids in treatment decision-making and monitoring therapeutic response over time.

The tool has been validated across various populations and languages and is recommended for routine use in both research and clinical settings to facilitate a standardised approach to evaluating postoperative bowel function.

Despite its simplicity, the LARS score does not capture all aspects of bowel dysfunction, particularly obstructive symptoms or their impact on daily functioning. To supplement this, other validated tools, such as the Memorial Sloan Kettering Bowel Function Instrument (MSKCC BFI) and the European Organisation for Research and Treatment of Cancer Quality of Life Questionnaire for Colorectal Cancer (EORTC QLQ-CR29), are often employed. These tools assess broader dimensions of bowel function and its psychological and social impacts [24]. Digital diaries, stool consistency scales, and anorectal manometry may provide further objective assessment in select patients.

Management strategies

Dietary Modifications

Dietary modifications serve as a first-line approach. Patients are advised to avoid triggers such as caffeine, spicy foods, and alcohol, and to increase their intake of fibre, such as methylcellulose [25].

Medications

Pharmacological treatments play a central role. Antidiarrheal agents like loperamide help reduce frequency and urgency by slowing colonic transit and increasing sphincter tone. 5-HT3 antagonists, such as ramosetron, are particularly effective in controlling urgency and postprandial symptoms [26]. Bulking agents and bile acid sequestrants, such as psyllium and colesevelam, respectively, can improve stool consistency and manage incontinence.

Transanal Irrigation

Transanal irrigation is a mechanical evacuation method that improves incontinence, urgency, and frequency [27].

Pelvic Floor Rehabilitation and Biofeedback

Pelvic floor rehabilitation and biofeedback are recommended for persistent symptoms beyond six months. These include Kegel exercises and sphincter training, which have been shown to improve continence and quality of life [28,29].

Sacral Nerve Stimulation (SNS)

SNS is an effective treatment for patients with refractory incontinence and urgency. It works through neuromodulation of the sacral nerves, helping to restore anorectal coordination. In extreme cases where quality of life is significantly impaired and conservative measures have failed, surgical options such as graciloplasty, neosphincter creation, or permanent colostomy may be considered [30].

Surgical Interventions

Surgical interventions, including sphincteroplasty or neosphincter creation, may be necessary in refractory cases. In severe, intractable LARS, a permanent colostomy may be considered [31].

Psychosocial Support

Psychosocial support remains integral throughout the treatment journey. Cognitive behavioural therapy, support groups, and the involvement of specialist nurses can alleviate anxiety, reduce social isolation, and empower patients in managing their symptoms.

Probiotics

Probiotics have limited evidence for efficacy, and more research is needed [32].

Prevention of LARS

Preventing LARS requires a proactive and multidisciplinary strategy that begins in the preoperative period. Preoperative patient counselling is essential to set realistic expectations and promote understanding of the potential risks of LARS. Patients should be informed about the possibility of postoperative bowel dysfunction, as well as the available management strategies.

Optimising surgical technique is paramount. Nerve-sparing total mesorectal excision (TME), preservation of the left colic artery to maintain perfusion, and construction of colonic J-pouches or transverse coloplasty may preserve neorectal compliance and reduce symptoms. Intraoperative nerve monitoring and meticulous dissection along anatomical planes can help minimise autonomic nerve injury.

Initiating pelvic floor rehabilitation preoperatively, known as prehabilitation, has shown potential in enhancing postoperative outcomes. Incorporating pelvic floor exercises and breathing techniques into preoperative care prepares patients for better functional recovery. Furthermore, Enhanced Recovery After Surgery (ERAS) protocols promote early mobilisation, early enteral nutrition, and minimised opioid use, all of which support gastrointestinal recovery.

Postoperative monitoring is equally critical. Structured follow-up with early identification of symptoms enables timely intervention. Patients should be assessed regularly for symptom evolution and functional impact, allowing dynamic adjustment of management strategies.

Emerging therapies and future direction

The management of LARS continues to evolve with novel therapeutic strategies under investigation. Modulation of the gut microbiome is gaining attention, as alterations in microbial diversity have been linked to postoperative bowel dysfunction. Probiotic supplementation and dietary prebiotics may have a role in restoring gut homeostasis, although evidence remains preliminary.

Transcutaneous tibial nerve stimulation (TTNS) has emerged as a less invasive alternative to SNS. Early studies have shown promise in improving continence and reducing urgency, offering an accessible and cost-effective modality for selected patients [33].

Digital health tools, including mobile applications and wearable technology, are being developed to monitor symptoms in real time and deliver personalised treatment recommendations. These tools enhance patient engagement, facilitate data collection for clinical decision-making, and support remote management.

Initiatives such as the LARS Consortium Assessment Tool (LARSCAT) and the Patient-Reported Outcomes Measurement Information System (PROMIS) are working to develop comprehensive assessment frameworks that incorporate both physical and psychosocial dimensions. These tools aim to standardise reporting and enable comparative research across populations.

Furthermore, regenerative medicine techniques, including stem cell therapy and tissue engineering, are under exploration as future avenues for restoring anorectal function. Although still in experimental stages, these innovations represent exciting possibilities for addressing the underlying pathophysiology of LARS.

## Conclusions

LARS has emerged as a significant postoperative challenge for patients undergoing sphincter-preserving surgery for rectal cancer. Despite advancements in surgical technique and neoadjuvant therapies, the high prevalence of LARS necessitates continued clinical attention. Its multifactorial pathophysiology, including neural, anatomical, and functional changes, underscores the complexity of this condition. Early identification and stratification of symptom severity through validated tools like the LARS score enable timely intervention. A stepwise, patient-centred management strategy, ranging from dietary adjustments to advanced modalities such as biofeedback therapy and sacral nerve stimulation, can meaningfully improve outcomes. Moreover, integrating psychological support and setting realistic expectations preoperatively are vital components of holistic care. Future research should focus on refining surgical techniques and expanding evidence-based therapies aimed at restoring bowel function and quality of life.
